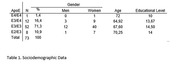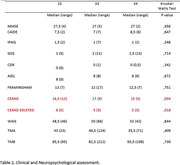# Cognitive characteristics in a Uruguayan cohort of healthy subjects with high educational level in risk of development dementia and its relation with ApoE genotype

**DOI:** 10.1002/alz.089667

**Published:** 2025-01-03

**Authors:** Francesca Mariani, Andres Damian, Alice Leites, Lucia Cortabarría, Laura Bocos, Sofia Santero, Valeria Contreras, Rodolfo Ferrando, Margarita García‐Fontes, Laura Reyes, Florencia Arredondo, Juan Andrés Abin‐Carriquiry, Mercedes Menendez, Alejandra Amestoy, Maren Torheim, Roberto Superchi, Ramon Suarez, Fabian Preciozzi, Laura Uriarte, Leandro Dansilio, Sergio Dansilio, Ana Charamelo, Ricardo Allegri, Paulo Caramelli, Francisco Lopera, Ricardo Nitrini, Gustavo Sevlever, Ana Luisa Sosa, Ismael Luis Calandri, Lucia Crivelli, Rosa Maria Salinas‐Contreras, Claudia Kimie Suemoto, Lina Marcela Vellila, Monica Sanches Yassuda

**Affiliations:** ^1^ Hospital de Clínicas, Montevideo, Montevideo Uruguay; ^2^ Centro Uruguayo de Imagenología Molecular, CUDIM., Montevideo, Montevideo Uruguay; ^3^ Atgen Srl., Montevideo, Montevideo Uruguay; ^4^ Hospital Británico, Montevideo, Montevideo Uruguay; ^5^ Instituto Universitario Asociación Cristiana de Jóvenes, ACJ., Montevideo, Montevideo Uruguay; ^6^ Fleni, Buenos Aires, Buenos Aires Argentina; ^7^ Behavioral and Cognitive Research Group, Faculdade de Medicina, Universidade Federal de Minas Gerais, Belo Horizonte Brazil; ^8^ Grupo de Neurociencias de Antioquia, Universidad de Antioquia, Medellin Colombia; ^9^ Universidade de Sao Pablo, Sao Pablo Brazil; ^10^ Dementias Laboratory, National Institute of Neurology and Neurosurgery, Mexico City, DF Mexico; ^11^ Fleni, Buenos Aires Argentina; ^12^ Fleni, Buenos Aires, CABA Argentina; ^13^ University of São Paulo Medical School, São Paulo, São Paulo Brazil; ^14^ University of São Paulo, SAO PAULO, SAO PAULO Brazil

## Abstract

**Background:**

ApoE has been linked to individual differences in risk and resilience to neurodegeneration in normal aging. The ApoE4 genotype has been associated with an increased risk of developing late‐onset Alzheimer’s disease (age 65 and older). Within the cognitively healthy population, important differences have been reported in the distribution of ApoE4 alleles and their association with cognitive performance, especially in underrepresented groups. We describe the distribution of ApoE alleles in healthy subjects at risk of developing dementia in Uruguay, and its association with cognitive assessment and risk factors for dementia.

**Method:**

we studied 73 subjects from the LatAm FINGERS project – Uruguayan cohort. Individuals had biobank analysis for the ApoE4 genotype, cognitive evaluation and risk factors for dementia. We compare the performance of participants in cognitive testing in relation with their ApoE allele characterization, and its relation with risk factors.

**Result:**

demographic characteristics of study subjects are shown in Table 1. We report only one participant with E4/E4 allele combination and was exclude of analysis groups. Kruskal‐Wallis test (Table 2) revealed a significant difference in CERAD and CERAD Delayed measures between the different APOE allele combinations. We didn´t observed differences in other cognitive parameters. Also, Spearman´s Correlation showed an association between educational level, CERAD and CERAD delayed, GDS and CDR. Framingham Risk score was significantly correlated with CAIDE score.

**Conclusion:**

our results suggest that the CERAD and CERAD Delayed may vary in different APOE allele combinations in normal subjects with a high educational level at risk of dementia in Uruguay. Understanding how genetic variability contributes to individual differences in cognition is important for the design of multidomain assessments and interventions, not only in subjects with cognitive impairment, but also in normal ageing in underrepresented populations.